# Worcester Lunatic Asylum

**Published:** 1845-06

**Authors:** 


### WORCESTER LUNATIC ASYLUM.

Dr. Edward Jarvis, who has for many years taken the liveliest interest in everything that relates to the insane, and to whom the profession owes some valuable papers on the Lunatic Asylums of the United States, has forwarded us the twelfth annual "Report of the Trustees of the State Lunatic Hospital, at Worcester," a document of great interest from which we shall make extracts for a future number. The institution for the insane, at Worcester, is one of the ornaments of our country and of the age. By the way, will not some friend at Lexington inform the public through our Journal what is the present condition of the Lunatic Asylum at that place? Frequent inquiries are made of us respecting its advantages, which we are not able to answer as definitely as we could desire. Y.

